# Understanding the formation mechanism and structural aspects of anti-cancer drug platinum uracil blue by quantum chemical studies

**DOI:** 10.1039/d5ra08093b

**Published:** 2025-11-28

**Authors:** Avishek Ghatak, Snehasis Banerjee

**Affiliations:** a Department of Chemistry, Chandernagore College Strand Road, Bara Bazar, Chandannagar Hooghly West Bengal 712136 India; b Department of Chemistry, Hooghly Mohsin College Chuchura Hooghly West Bengal 712101 India sbanchem@gmail.com

## Abstract

The mechanism of the reaction between the hydrolysis product of cisplatin ([Pt(NH_3_)_2_(OH_2_)_2_]) and uracil leading to the formation of di- and tetra-valent platinum complexes with varying oxidation states, such as Pt(2+), Pt(2.25+), Pt(2.5+), and Pt(3+), was investigated using density functional theory (DFT). The formation of the dimer of *cis*-[Pt_2_(NH_3_)_4_(Ur)_2_]^2+^ (Ur = deprotonated uracil) from *cis*-[Pt(NH_3_)_2_(OH_2_)_2_]^2+^ proceeded through a four-step mechanism. In the first and second steps, two water molecules were successively replaced by two Ur to form *cis*-[Pt(NH_3_)_2_(Ur)_2_]. In the third step, *cis*-[Pt(NH_3_)_2_(Ur)_2_] dimerised to the diplatinum complex [Pt_2_(NH_3_)_4_(OH_2_)(Ur)(µ-Ur)]^2+^ using one exocyclic oxygen of uracil *via* a bridged bond, which subsequently formed [Pt_2_(NH_3_)_4_(µ-Ur)_2_]^2+^ in the fourth step. Pentacoordinated platinum transition states (TSs) and other stationary points on the potential energy surface were optimized and characterized. In the gas phase, the activation energy barriers for the formation of the head-to-head orientation increased progressively across the reaction steps. In contrast, in the solvent phase, although the barriers were generally comparable, the clear stepwise progression observed in the gas phase was not maintained. Time-dependent DFT (TD-DFT) was employed to examine the spectral changes from monomer to dimer to tetramer forms. Intermolecular interactions in tetramers were characterized using reduced density gradient (RDG). Furthermore, quantum theory of atoms in molecules (QTAIM) analysis was employed to compare the binding character of Pt(2+)_4_ and Pt(2.25+)_4_.

## Abstract 2

DFT and TDDFT studies reveal the stepwise hydrolysis and uracil-assisted dimerization of cisplatin, forming di- and tetra-valent Pt complexes through Pt–O–Pt bridging pathways.

## Graphical abstract

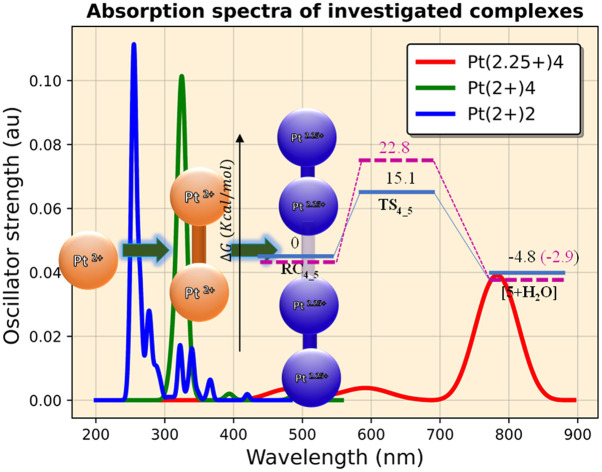

## 1. Introduction

Although cisplatin^1^ or *cis*-DDP ([Pt(NH_3_)_2_Cl_2_]) was effective and successful against various types of cancer, its side effects encouraged researchers to design new classes of Pt-drugs with improved anti-cancer properties. A family of deeply coloured platinum compounds, usually called ‘platinum blues’, has attracted the interest of researchers because of its unusual colour and high antitumor activities.^2,3^ Unlike the typical yellow, orange, red, or colourless platinum complexes, platinum blues are distinguished by their striking blue or purple colours.

Structural evidence for platinum blues was first obtained through single-crystal X-ray analysis of the ‘α-pyridonate blue’ complex, where ‘α-pyridone’ served as a simplified model for pyrimidine bases.^4,5^ As a result, numerous homo- and mixed-valence di- and tetra-nuclear platinum complexes have been successfully isolated and structurally characterized.^6–14^ Crystallographic studies have confirmed that the basic structure of the Pt–pyrimidine blues is defined by a tetranuclear zigzag chain structure, comprising two dinuclear Pt units with bridging amidate ligands.^6^ These mixed-valent complexes contain three Pt(ii) ions and one Pt(ii) ion and are paramagnetic due to the presence of unpaired electrons. Platinum blues are primarily classified based on the oxidation states of Pt and the orientation of the bridging amidate ligands within the dimeric unit. Furthermore, Pt(ii) dimers can undergo a two-electron oxidation to produce Pt(iii) species,^6^ which may also be generated through the reduction of platinum blue intermediates under specific conditions. Lippert *et al.* studied the formation of platinum [Pt_2.25_]^4^-1-methyluracil blue through silver(i) oxidation of [Pt_2.0_]^2^ and isolated a heteronuclear (Pt_2_, Ag_2_) precursor to investigate the origin and structural features of “platinum uracil blue” (PUB).^10^

**Scheme 1:**
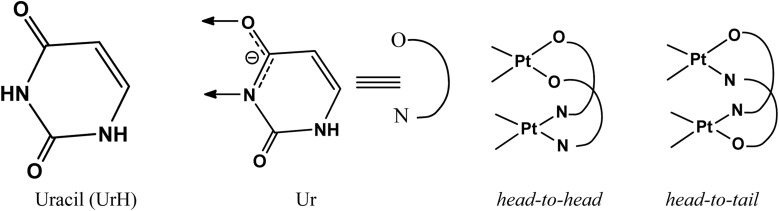
Uracil pyrimidine and its deprotonated form, along with the two configurations of the dimeric structure.

Each dimeric structure incorporates two bridging uracil ligands, which can adopt one of the two configurations of head-to-head (h–h), where one platinum atom binds to two uracil ring nitrogens and the other coordinates with two exocyclic oxygens, or head-to-tail (h–t), where both platinum atoms coordinate with one nitrogen and one oxygen (Scheme 1). The steric bulk of the exocyclic amidate rings at both the ends of the unit prevents the formation of tetramers from h–t dimers.^15^ In contrast, the oxidation of h–h dimers following dimerization results in the formation of the core tetrameric structure.

Despite extensive experimental efforts to synthesize platinum pyrimidine blues (PPBs) and elucidate their chemical features, no studies have yet explored the reaction mechanism of their formation.^16^ Detailed theoretical computations are crucial to understanding the synthetic mechanism, the causes of the deep blue coloration, and the interactions present in polynuclear homo- and mixed-valent platinum complexes.

**Scheme 2:**
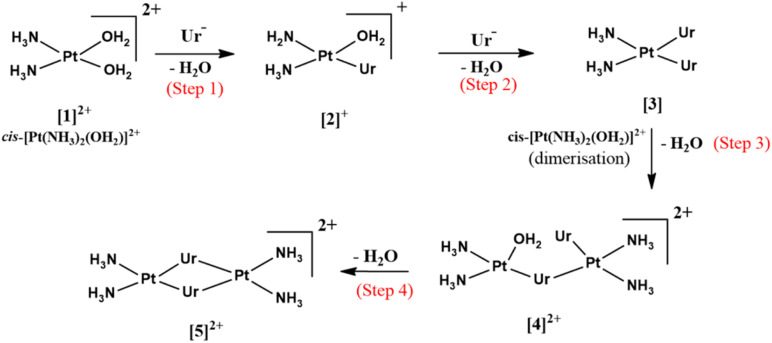
Schematic of the formation mechanism of Pt(2+)_2_ complexes from the hydrolysis product of *cis*-DDP.

In this study, we investigated the mechanism behind the formation of platinum–uracil dimer complexes from the hydrolysis product of *cis*-DDP in detail using computational chemistry (Scheme 2).

As the bridged Pt–Pt distance is a critical parameter for catalytic activity, where shorter Pt–Pt distances are often correlated with an enhanced catalytic performance, we examined the structural aspects of various di- and tetra-meric platinum–uracil complexes across four oxidation states (2+, 2.25+, 2.5+, and 3+).^16^ This analysis included both intra- and inter-molecular Pt–Pt distances.

To explore the intermolecular stabilizing forces that drive the formation of tetramers from dimeric Pt complexes and to analyse the non-covalent interactions, tetranuclear Pt complexes were studied in the Pt(2+) and Pt(2.25+) oxidation states using QTAIM and reduced density gradient (RDG) calculations. Additionally, spin density measurements were conducted to assess the localization or delocalization of unpaired electrons.

The spectral properties of these di- and tetra-nuclear platinum–uracil compounds were also calculated and analyzed to understand their color characteristics. Furthermore, the stabilizing role of an axial donor, specifically chloride, was investigated to evaluate its impact on complex stability.

## 2. Computational details

The geometrical parameters of all the chemical species were calculated using restricted or unrestricted approximations of the Kohn–Sham equations, depending on the total electron count.

A benchmark study was carried out on complex 3 utilizing the B3LYP,^17^ M06-2X,^18^ PBE0,^19^ wB97X-D (ref. 20) and B3LYP-GD3BJ functionals.^21^ The 6-311+G(d) triple-ζ split-valence basis set was employed for the light atoms (H, C, N, and O), while the transition-metal center (Pt) was described using the Stuttgart/Dresden (SDD) effective core potential. A comparison with experimental data confirmed that the wB97X-D functional, in combination with these basis sets, provided the best results. This level of theory yielded a small mean unsigned error (MUE) of 0.041 Å for bond lengths and 1.6° for bond angles, as summarized in Table 1. Although B3LYP was used for the preliminary initial guess (B3LYP/SDD/6-31G(d)) and exhibited a relatively small MUE (0.053 Å for bond lengths and 2.7° for bond angles), it is frequently reported as a suboptimal choice in various studies.^22–24^ Consequently, the wB97X-D/SDD/6-311+G(d) combination was selected for all further investigations. All calculations were carried out using the Gaussian 09 program package, while Gaussian 16 was employed for computations utilizing the B3LYP-GD3BJ functional.^25^

**Table 1:** Unsigned error and mean unsigned error (both in Å) computed for 3, employing different functionals, with respect to the experimental values^a^

Bond distances and bond angles	Exp.^b^ (Å)	Calc.^c^ (Å)				
		B3LYP	M06-2X	PBE0	wB97X-D	B3LYP-GD3BJ
Pt–N_Ur_1__	2.041	0.033	0.018	0.009	0.020	0.025
Pt–N_Ur_2__	2.034	0.04	0.053	0.018	0.025	0.031
Pt–N_am_1__	2.019	0.082	0.084	0.053	0.070	0.076
Pt–N_am_2__	2.042	0.057	0.069	0.031	0.048	0.053
N_Ur_1__–Pt–N_Ur_2__	90.4	2.3	0.2	1.2	0.4	0.4
N_Ur1_–Pt–N_am_1__	175.5	2.3	1.8	2.1	1.7	1.8
N_Ur_1__–Pt–N_am_2__	89.4	3.1	2.3	2.7	2.4	2.3
N_Ur_2__–Pt–N_am_1__	89.7	3.2	2.9	2.8	0.9	2.9
N_Ur_2__–Pt–N_am_2__	176.3	1.5	1.4	1.2	0.1	1.2
N_am_1__–Pt–N_am_2__	90.7	3.8	3.6	3.9	4.5	3.6
MUE (bond distances)		0.053	0.056	0.028	0.041	0.046
MUE (bond angles)		2.7	2.0	2.3	1.6	2.0

a MUE = mean unsigned error is the average of all.

b Ref. 8.

c In all the cases, SDD/6-311+(d) basis were used.

No symmetry constraints were applied during the optimization process. The geometries of the intermediates and reactant/product complexes were confirmed as true minima on the potential energy surface (PES) by the absence of any imaginary frequencies. Conversely, the transition state (TS) geometries were confirmed as first-order saddle points on the PES by exhibiting only one imaginary frequency. The reliability of the TS structures was further verified by computing the intrinsic reaction coordinates (IRC) at the same level of theory.

To obtain more accurate thermodynamic data, the low-frequency vibrational modes were treated using the quasi-rigid rotor harmonic oscillator (quasi-RRHO) model instead of the standard RRHO (harmonic approximation). The energy values were subsequently recalculated using the Shermo 2.6 program.^26^

The solvent effect was incorporated using the polarizable continuum model (PCM),^27^ the default solvation model in Gaussian 09, at the wB97X-D level with the same basis set combination. While mononuclear platinum complexes were optimized directly in the solvent media, convergence was proved difficult for diplatinum complexes due to extensive time requirements and fluctuations in the results. For the latter, a single-point energy calculation in the solvent was used, corrected by the gas-phase zero-point energy. We optimized a representative dinuclear Pt complex in PCM at the same level of theory used throughout the study. To assess the effect of solvent-driven structural relaxation, we performed a full geometry optimization in the solvent (PCM) for a representative dinuclear Pt complex at the same level of theory used in the study. Table S1 shows the selected Pt–ligand distances and key structural parameters from gas-phase and PCM-optimized geometries. The mean unsigned error (MUE) between gas-phase and PCM-optimized bond lengths was 0.021 Å. The largest individual change was Pt_2_–O_wat_ = 0.118 Å (a flexible solvent ligand), while Pt–Pt changed by 0.077 Å, and the O_wat_–Pt_2_–O_Ur_2__ angle changed by only 2°. The PCM-optimized geometry was lower in energy by ≈0.64 kcal mol^−1^ than the gas-phase structure, indicating minor stabilization owing to the small solvent-induced relaxation. These small differences indicated that solvent relaxation did not significantly alter the geometry or the energetics of the system; therefore, the use of single-point PCM corrections on gas-phase optimized structures was adequate for the comparisons and the time-dependent density functional theory (TD-DFT) spectra reported in this work.

The TD-DFT method was employed in media to study the electronic transitions and determine the theoretical absorption spectra based on the equilibrium ground state geometry (*S*_0_). The solvent was included *via* the PCM. TD-DFT calculations were carried out to obtain the lowest 25 excited states, with the self-consistent field (SCF) convergence threshold set to 10^−8^ a. u. For open-shell doublet systems, the 〈*S*^2^〉 values were monitored to assess the spin contamination. The computed 〈*S*^2^〉 values were typically around 0.757, which was very close to the ideal value of 0.75, confirming a negligible spin contamination.

The activation energy barrier is defined as the Gibbs free energy difference between the transition state and the reactant complex, representing the kinetic feasibility of a reaction step. The reaction free energy (Δ*G*) is the Gibbs free energy difference between the products and reactants, reflecting the thermodynamic spontaneity of the process.

To check the stacking and hydrogen bonding interactions between the ammine hydrogen and the amidate oxygen atoms, we employed non-covalent interaction (NCI) and quantum theory of atoms in molecules (QTAIM) using Multiwfn.^28^ Similar methodologies were utilized in some of our previous works.^29–36^

## 3. Results and discussion

### 3.1 Pathway of the formation of *cis*-[Pt(NH_3_)_2_(Ur)_2_] (Ur = uracil) from *cis*-[Pt(NH_3_)_2_(H_2_O)_2_]

The formation of *cis*-[Pt(NH_3_)_2_(Ur)_2_] ([3]) from the hydrolysis product of cisplatin, *cis*-[Pt(NH_3_)_2_(H_2_O)_2_]^2+^ ([1]^2+^), proceeds through a two-step mechanism. In the first step, one water molecule is replaced by a deprotonated uracil (Ur) molecule to form *cis*-[Pt(NH_3_)_2_(Ur)(H_2_O)]^+^ ([2]^+^), which then reacts with the deprotonated nitrogen of another Ur molecule to form *cis*-[Pt(NH_3_)_2_(Ur)_2_] in the second step.

The nomenclature of molecular species follows the convention described as follows: for example, RC_1_2_ denotes the reactant complex formed prior to the transition state TS_1_2_, while PC_1_2_ represents the product complex formed after the decomposition of TS_1_2_. Similarly, subsequent intermediates and transition states (RC_3_4_, TS_4_5_, *etc.*) are labeled consistently throughout this study.

**Fig. 1:**
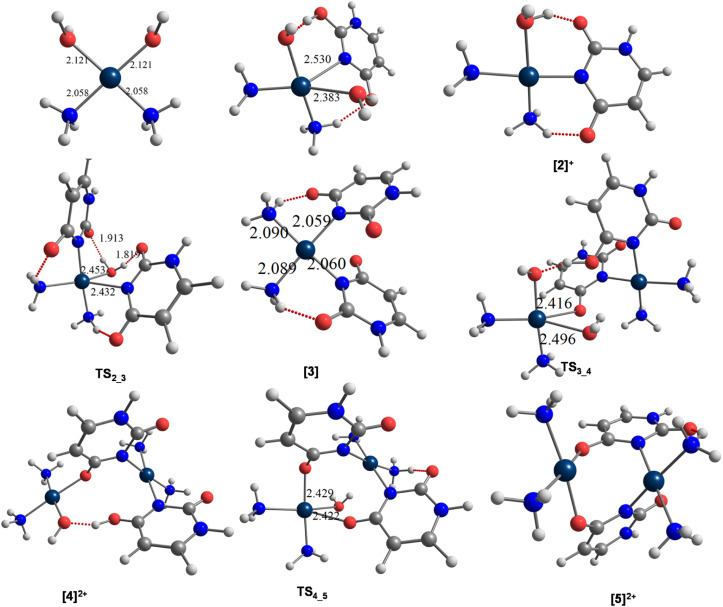
Optimized geometries of species involved in the reaction between the hydrolysis product of *cis*-DDP [1]^2+^ and uracils, leading to the formation of diplatinum complex [5]^2+^ (Pt(2+)_2_), computed at the wB97X-D/SDD/6-311G+(d) level of theory.

The conversion of [1]^2+^ to [2]^+^ in the first step occurs *via* a trigonal-bipyramidal (tbp) transition state TS_1_2_, characterized by an imaginary frequency of −156*i* cm^−1^. In TS_1_2_, the central Pt atom forms Pt⋯N (Ur) and Pt⋯O (water) bonds with incoming and leaving groups, with bond lengths of 2.383 Å and 2.529 Å, respectively. IRC calculations suggest that TS_1_2_ connects the reactant complex (RC_1_2_) and the product complex (PC_1_2_), with both structures stabilized by strong hydrogen bonds, as shown in Fig. 1 and S1.

**Fig. 2:**
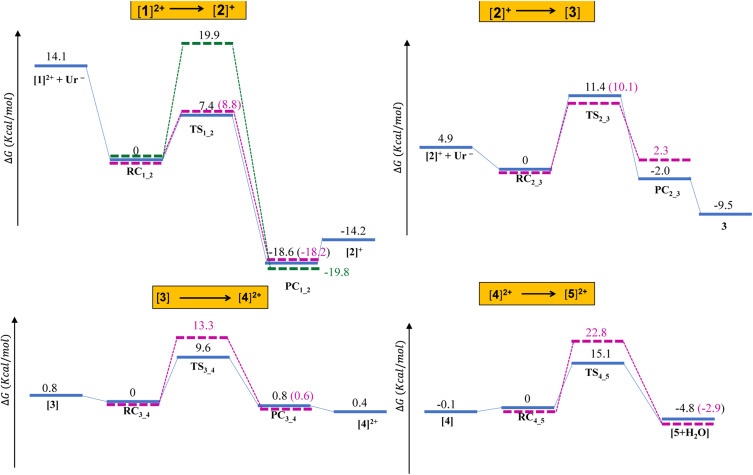
Energy diagram illustrating the formation of the diplatinum complex from the hydrolysis product of *cis*-DDP, calculated at the wB97X-D/SDD/6-311+G(d) level of theory. The different steps are highlighted in yellow. The diagram is not drawn to scale. Values in magenta correspond to gas-phase data. Values in green correspond to the nucleophilic attack by the oxygen atom of uracil.

The thermodynamic and kinetic parameters are presented in Table 2 and the energy diagram (Fig. 2). Results indicate that the first step involves a low energy barrier of 7.4 kcal mol^−1^ relative to the reactant complex RC_1_2_, which is stabilized by 14.1 kcal mol^−1^ compared with the isolated [1^2+^] complex and the uracil anion (Ur^−^) (Table 2). This stability is likely due to the formation of a complex from two oppositely charged species. The reaction is highly exothermic (Δ_r_*G* ≈ −18.6 kcal mol^−1^, Δ_r_*H* ≈ −18.0 kcal mol^−1^), making this step both thermodynamically and kinetically favourable.

**Table 2:** Thermodynamic and kinetic parameters involved in the investigated transformation computed at the wB97X-D/SDD/6-311+G(d) level of theory in solvent. All values are in kcal mol^−1^

Reaction steps		Δ*E*^a^	Δ*G*^a^	Δ_r_*H*
Step 1	N-Donor	6.57	7.14	6.85
	O-Donor	19.9	19.8	−20.4
Step 2		9.76	11.45	9.38
Step 3		11.09	9.67	9.95
Step 4		16.30	15.8	15.2

a Represents activation energy barrier.

To form the head-to-tail dimer, initial binding *via* the exocyclic oxygen of uracil is essential. An alternative pathway involving oxygen as the donor was also investigated. However, this route requires a much higher activation energy compared with the N-donor binding. The optimized geometries of RC_1_2_(O), TS_1_2_(O), PC_1_2_(O) and [2(O)]^+^ are shown in Fig. S2. The first step, where the oxygen of uracil acts as a nucleophile, exhibits a significantly higher activation energy barrier of 19.9 kcal mol^−1^.

In the second step, [2]^+^ forms [3], reacting with a second uracil anion *via* another trigonal-bipyramidal transition state TS_2_3_, characterized by an imaginary frequency of −168*i* cm^−1^. In TS_2_3_, the central Pt atom forms Pt⋯N (Ur) bond with the second uracil molecule at a distance of 2.431 Å as well as a Pt⋯O (water) bond with the departing water molecule, which exhibits a slightly elongated bond length of 2.452 Å. IRC calculations suggest that TS_2_3_ connects the reactant complex (RC_2_3_) and the product complex (PC_2_3_). The reactant complex RC_2_3_ is highly stabilized by strong hydrogen bonds, as shown in Fig. 1 and S1. In RC_2_3_, the leaving water molecule occupies the axial position with a Pt⋯O distance of 3.156 Å.

In this step, the formation of [3] from [2]^+^ occurs with an activation energy barrier of 11.4 kcal mol^−1^, calculated relative to the reactant complex RC_2_3_. RC_2_3_ is highly stabilized (4.9 kcal mol^−1^) compared with the isolated reactants ([2]^+^ and Ur^−^). This reaction step is slightly endothermic, with free energy changes of 2.0 kcal mol^−1^ and 9.5 kcal relative to the product complex PC_2_3_ and [3], respectively.

### 3.2 Pathway of the formation of diplatinum N,O-bridged Pt(ii) complex [Pt_2_(NH_3_)_4_(Ur)_2_]^2+^ from *cis*-[Pt(NH_3_)_2_(Ur)_2_]

As stated, diplatinum(ii) complex [Pt_2_(NH_3_)_4_(Ur)_2_]^2+^ may be oriented in two ways: h–h or h–t, as shown in Scheme 1. The formation of both the dinuclear complexes, h–h and h–t [Pt_2_(NH_3_)_4_(Ur)_2_]^2+^, through the reaction of another *cis*-[Pt(NH_3_)_2_(H_2_O)_2_] unit involves two steps.

#### 3.2.1 Formation of head-to-head oriented [Pt_2_(NH_3_)_4_(Ur)_2_]^2+^

In the first step, a Pt⋯Ur⋯Pt bridge bond is formed through the reaction of [3] with [1]^2+^*via* a trigonal bipyramidal transition state TS_3_4_, characterized by an imaginary frequency of −168*i* cm^−1^ (Fig. 1). The trigonal plane of the tbp transition state consists of Pt⋯O (water) and Pt⋯O (first Ur) bonds with bond lengths of 2.496 Å and 2.415 Å, respectively. TS_3_4_ is stabilized by strong hydrogen bonds between the two mononuclear platinum units, along with a proton transfer from the axial second water molecule of [1]^2+^ to the ketonic bond of the second Ur molecule in [3]. IRC calculations reveal that TS_3_4_ connects the reactant complex RC_3_4_ and the product complex PC_3_4_. Both complexes are stabilized by some hydrogen bonds. PC_3_4_ undergoes conversion to [4]^2+^. This step proceeds through an energy barrier of 9.6 kcal mol^−1^ relative to the reactant complex RC_3_4_ and exhibits slight endothermicity, with a net energy change of 0.8 kcal mol^−1^ compared with the product complex PC_3_4_.

In the second step, [4]^2+^ transforms into [5]^2+^*via* a trigonal-bipyramidal transition state, TS_4_5_ (−154.7*i* cm^−1^). In TS_4_5_, the trigonal plane includes the Pt⋯O(water) and Pt⋯O(uracil) interactions, with corresponding distances of 2.432 Å and 2.442 Å, respectively. This transition state connects the reactant complex (RC_4_5_) and the product diplatinum(ii) ring complex [Pt_2_(NH_3_)_4_(Ur)_2_]^2+^ ([5]^2+^), formed through the elimination of a water molecule and the creation of two bridging bonds *via* the uracil ligands. In [5]^2+^, the Pt⋯Pt distance is further reduced to 3.305 Å.

This final step, involving the formation of Pt(2+)_2_ through bridging bonds, has a moderately high energy barrier of 15.1 kcal mol^−1^ relative to the reactant complex RC_4_5_. This step is slightly exothermic, with an energy change of −4.8 kcal mol^−1^ relative to RC_4_5_.

### 3.3 Non-covalent interaction (NCI) and quantum theory of atoms in molecules (QTAIM)

We computed the reduced density gradient (RDG) to represent the deviation from a homogeneous electron distribution (eqn (1)):1
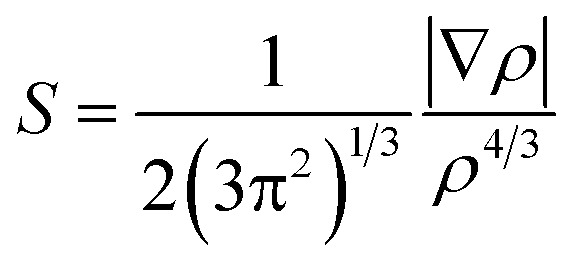
where ∇ is the gradient operator, and |∇*ρ*| is the electronic density gradient mode, offering a powerful approach to explore and visualize various types of non-covalent interactions (NCIs) in real space, including both intra- and inter-molecular weak interactions such as hydrogen bonds and van der Waals forces. The NCI index is therefore employed to analyze the non-covalent interactions within the multinuclear platinum complexes.

**Fig. 3:**
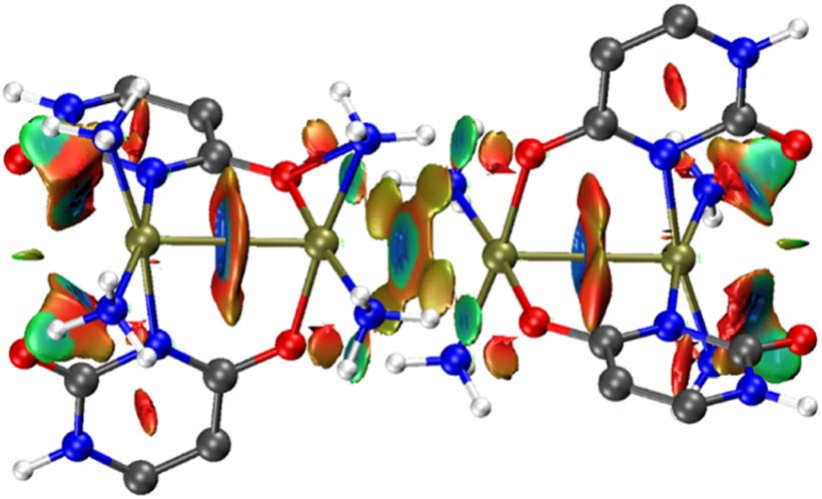
NCI plot index analysis for Pt(2+)_4_ at the B3LYP/SDD/6-31G(d) level of theory. The NCI index isosurface is 0.5 a. u. The density cut-off is 0.03 a. u., and the colour code has the usual significance.

The color-mapped isosurfaces of the investigated tetramer complexes are depicted in Fig. 3. In these RDG (reduced density gradient) isosurfaces, blue regions denote hydrogen bonds, green areas highlight van der Waals interactions, and red regions indicate steric hindrance. The intensity of each color corresponds to the interaction strength, with darker shades representing stronger interactions.

The presence of four hydrogen bonds, indicated by blue patches, contributes significantly to the stabilization of the tetramer [Pt(NH_3_)(Ur)_2_].^2^ Additionally, the Pt–Pt intradimer non-covalent interactions are highlighted by green patches, demonstrating their role in stabilizing the structure.

The quantum theory of atoms in molecules (QTAIM), introduced by Bader and collaborators is a widely used approach in computational chemistry to analyze molecular interactions and bond strengths.^37^ This analysis relies on several critical parameters, including the Laplacian of the electron density (∇^2^*ρ*(*r*)), kinetic energy density (*G*(*r*)), potential energy density (*V*(*r*)), and their ratio (−*G*(*r*)/*V*(*r*)). Among these, the electron density (*ρ*(*r*)) and its Laplacian (∇^2^*ρ*(*r*)) are particularly important for characterizing the nature of bonds or interactions.

**Fig. 4:**
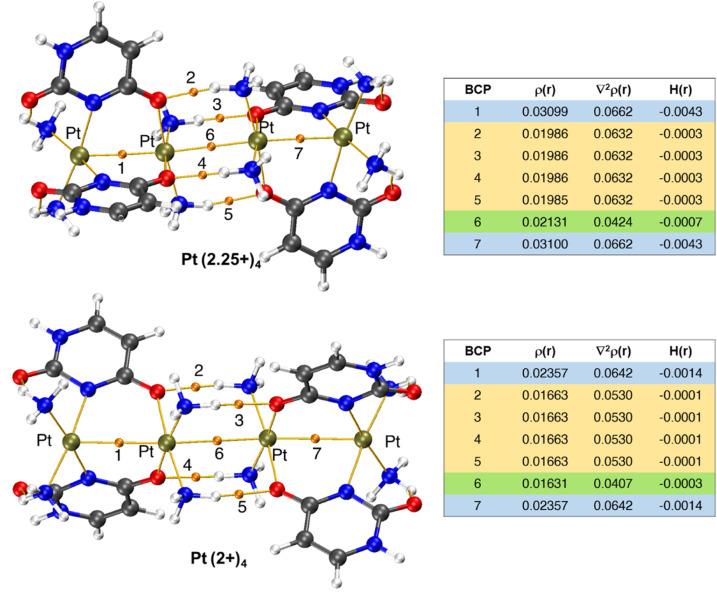
QTAIM distribution of BCPs (orange spheres) and bond paths in the Pt(2+)_4_ and Pt(2.25+)_4_ complexes. Only relevant CPs are shown for clarity.

A high electron density (*ρ*(*r*) > 0.20 a. u.) combined with negative values of ∇^2^*ρ*(*r*) indicates a covalent bond. In contrast, a low electron density (*ρ*(*r*) < 0.10 a. u.) and positive ∇^2^*ρ*(*r*) are characteristic of closed-shell interactions, such as ionic bonds, hydrogen bonds, or van der Waals forces. Hydrogen bonds can be further classified based on ∇^2^*ρ*(*r*) and total energy density (*H*(*r*)). Weak hydrogen bonds exhibit ∇^2^*ρ*(*r*) > 0 and *H*(*r*) > 0, moderate hydrogen bonds are characterized by ∇^2^*ρ*(*r*) > 0 and *H*(*r*) < 0, and strong hydrogen bonds display ∇^2^*ρ*(*r*) < 0 and *H*(*r*) < 0. Moreover, approximate binding energy can be calculated using the expression provided by Espinosa *et al.*: BE = *V*(*r*)/2.^38^ In Fig. 4, we have shown seven relevant BCPs, namely, for Pt–Pt (intra dimer), Pt–Pt (inter dimer), and four O–H bonds. Results show that all these CPs are involved in weak interactions. In both the tetramers, two dimeric units are stabilised by one Pt–Pt bond and four hydrogen bonds between NH_3_ of one dimer and the exocyclic oxygen of another dimer. The *ρ*(*r*) values show that these hydrogen bonds are stronger in Pt(2+)_4_ than that in Pt(2+)_2_. Additionally, it should be noted that as the Pt(2+)_2_ unit forms the Pt(2.5+)_4_ dimer through oxidation, all five main bonds—four hydrogen bonds and one interdimer Pt–Pt bond—become stronger, enhancing the stability of the mixed-valence complex.

### 3.4 FMOs and time-dependent density functional study

**Fig. 5:**
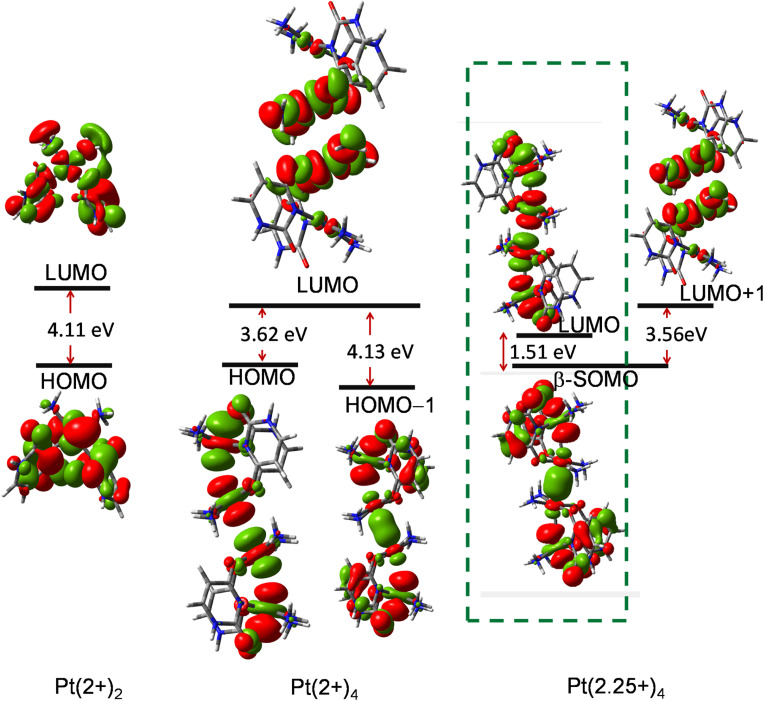
FMOs of Pt(2+)_2_, Pt(2+)_4_, and Pt(2+)_4_.

To understand the deep blue coloration of mixed-valence platinum complexes, we computed theoretical absorption spectra using the TD-DFT method and frontier molecular orbital (FMO) analysis. The FMOs, particularly the HOMO and LUMO, play crucial roles in determining the optical and electronic properties. Fig. 5 presents the HOMO/LUMO energy levels and their gaps for Pt(2+)_2_, Pt(2+)_4_ and Pt(2.25+)_4_ complexes.

Results show that the HOMO–LUMO gap decreases in the following order: 4.11 eV (Pt(2+)_2_) > 3.62 eV (Pt(2+)_4_) > 1.51 eV (Pt(2.25+)_4_, β-SOMO–β-LUMO). This trend indicates that the Pt(2.25+)_4_ complex has the lowest kinetic stability and the highest reactivity.^39–41^ Moreover, electronic transitions become more facile from Pt(2+)_2_ to Pt(2+)_4_ and to Pt(2.25+)_4_ due to the progressive reduction in the optical gap.^41^

In Pt_4_^2+^, both σ and σ* orbitals are occupied as HOMO and HOMO−1, respectively. In contrast, in Pt(2.25+)_4_, the SOMO is localized on the intra-dimer σ_Pt–Pt_ bond, while the LUMO is positioned on the intra-dimer 
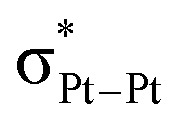
 bond.

**Fig. 6:**
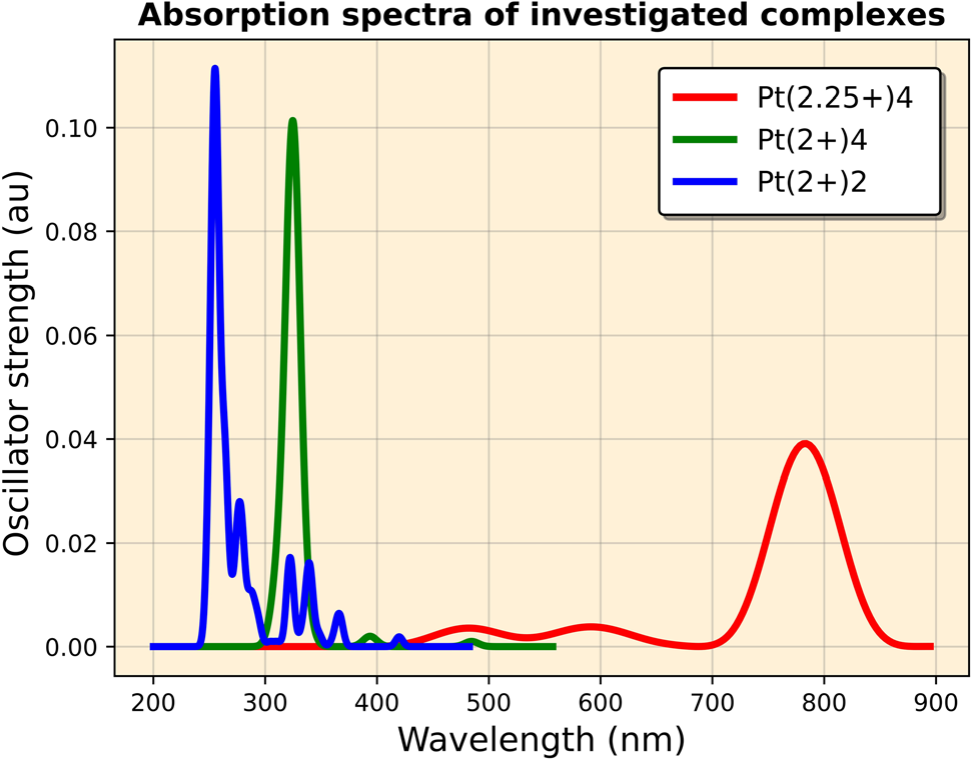
Calculated absorption spectra of Pt(2+)_2_, Pt(2+)_4_, and Pt(2+)_4_ in water.

TD-DFT calculations were performed based on the optimized ground-state geometry (*S*_0_) to eliminate the ghost states with artificially low excitation energies. The absorption data show that in Pt(2+)_2_, the lowest-energy absorption occurs at 420 nm (low oscillator strength, *f* = 0.001), primarily (87%) attributed to the HOMO → LUMO transition. However, more substantial absorption peaks are observed around 250 nm (Fig. 6).

For Pt(2+)_4_, the lowest-lying absorption appears at 483 nm, mainly contributed by the HOMO−1 → LUMO transition (80%). While no ghost states are detected for Pt(2+)_2_ and Pt(2+)_4_, the open-shell configuration results in some unphysical low-energy excitations (>1000 nm).^42,43^ However, for comparison with experimental results, we considered only the excitations below 800 nm (*f* = 0.001). Stronger peaks were found around 325 nm (*f* = 0.081).

For Pt(2.25+)_4_, an absorption peak appears around 790 nm (*f* = 0.04), in excellent agreement with available experimental results.^10^ This peak is associated with the electron transfer from the intra-dimer Pt–Pt σ orbital to the Pt–Pt σ* orbital. The mean unsigned error (MUE) values demonstrate good agreement between theory and experiment, particularly for the wB97X-D functional (MUE = 18 nm), thereby confirming the reliability of our computational approach.

The observed deviations are well within the expected accuracy range of TDDFT methods (typically ±20–30 nm), supporting that the chosen functional-basis set combination adequately reproduces the experimental spectra (Table S2).

## 4. Summary and conclusions

The reaction mechanism for the formation of tetraplatinum complexes [Pt_2_(NH_3_)(Ur)_2_] from the hydrolysis product of *cis*-DDP is investigated theoretically using DFT. Results reveal that this transformation occurs *via* a four-step mechanism.

In the first step, a single H_2_O molecule is replaced by deprotonated uracil through its ring nitrogen, forming [Pt(NH_3_)_2_(OH_2_)(Ur)]^1+^. In the second step, this intermediate further reacts with another ring nitrogen of deprotonated uracil, leading to the formation of [Pt(NH_3_)(Ur)_2_]. In the third step, the uracil complex [Pt(NH_3_)(Ur)_2_] reacts with another [Pt(NH_3_)_2_(OH_2_)_2_], forming the diplatinum complex [Pt_2_(NH_3_)_4_(OH_2_)(Ur)(µ-Ur)]^2+^. Here, the second *cis*-Pt(NH_3_)_2_ unit bridges through the ring nitrogen and exocyclic oxygen of one uracil ligand. Finally, in the fourth step, a head-to-head dimer is formed *via* two bridged bonds involving the ring nitrogen and exocyclic oxygen of both uracils.

The activation energy barriers increase progressively across the steps in the gas phase: 8.8 kcal mol^−1^ (step 1), 10.1 kcal mol^−1^ (step 2), 13.3 kcal mol^−1^ (step 3), and 22.5 kcal mol^−1^ (step 4) for the formation of the head-to-head orientation. In the solvent medium, the corresponding values are 7.4, 11.4, 9.6, and 15.1 kcal mol^−1^, respectively; however, a clear progressive trend is not observed in the solvent environment. Notably, the reaction involving the exocyclic oxygen in the first step (necessary for a head-to-tail orientation) has a much higher activation energy barrier of 19.9 kcal mol^−1^. Furthermore, head-to-tail tetramers were not observed due to steric hindrance caused by the exocyclic amidate rings at both the ends of the dimer unit.

Given the significance of Pt–Pt bond lengths in these complexes, a series of model di- and tetra-platinum complexes in head-to-head and head-to-tail orientations were studied in oxidation states 2+, 2.25+, 2.5+, and 3+.

The QTAIM analysis and NCI index calculations indicate that the tetra-Pt complexes achieve stability through four hydrogen bonds and an interdimer Pt–Pt bond. Furthermore, the analysis reveals that as the Pt(2+)_2_ unit undergoes oxidation to form the Pt(2.5+)_4_ dimer, all five primary bonds—four hydrogen bonds and one interdimer Pt–Pt bond—strengthen, thereby enhancing the stability of the mixed-valence complex.

Spin density values and plots for these complexes reveal that unpaired electrons are delocalized across the four platinum centers. The axial capping ligands are essential in influencing both the spin density and the overall stability of the mixed-valence complexes. We believe that this detailed study will be valuable for future researchers in understanding the properties of platinum uracil blue.

## Conflicts of interest

There are no conflicts to declare.

## Supplementary Material

RA-015-D5RA08093B-s001

## Data Availability

The Cartesian coordinates of all relevant species examined in this study are provided in the Supplementary information (SI). Selected optimized geometries are also included. Supplementary information is available. See DOI: https://doi.org/10.1039/d5ra08093b.
