# Supplementary material for: Understanding the formation mechanism and structural aspects of anti-cancer drug platinum uracil blue by quantum chemical studies
Source: RSC Adv. 2025 Nov 28;15(55):46914–24. doi: 10.1039/d5ra08093b (PMC12661138; doi:10.1039/d5ra08093b)
Supplement: RA-015-D5RA08093B-s001 [file RA-015-D5RA08093B-s001.pdf]

Supplementary Information (SI) for RSC Advances.
This journal is © The Royal Society of Chemistry 2025

Supporting Information

Understanding the Formation Mechanism and Structural Aspects of Anti-cancer Drug Platinum Uracil Blues by Quantum Chemical Studies

Avishek Ghatak,[a] Snehasis Banerjee[*,b]

[a] Department of Chemistry, Chandernagore College, Strand Road, Bara Bazar,Chandannagar, Dist: Hooghly, West Bengal, Pin: 712136, INDIA

[b] Department of Chemistry, Hooghly Mohsin College, Chuchura, P.O.- Chinsurah, Dist.-Hooghly,

West Bengal, India, 712101, INDIA

Figure S1: Optimized geometries of selected species involved in the investigated reactions

Figure S2: Optimized geometries of the transition state, reactant, and product complexes involved in the reaction between the oxygen atom of uracil in the first step of the investigated reaction

Table S1 Unsigned Error and Mean Unsigned Error Computed for **TS4_5** Employing Different Functional with Respect to the Experimental Values

Table S2 Unsigned Error and Mean Unsigned Error Computed for Pt(2.25+)$_4$ Employing Different Functional with Respect to the Experimental Values

Table S3: Cartesian coordinates of the species involved at wB97X-D/SDD/6-311G+(d) in the investigated reactions

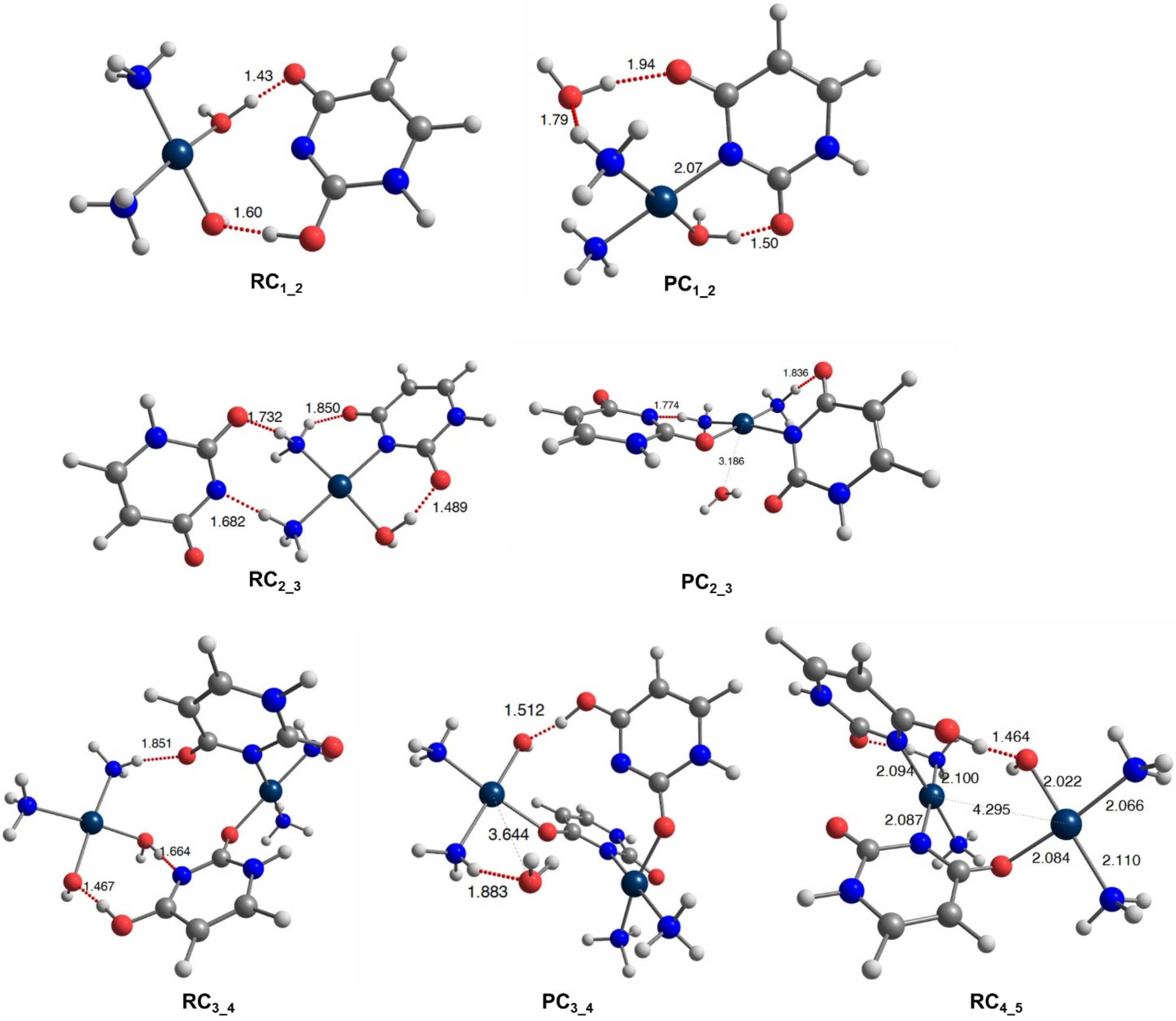

Figure S1: Optimized geometries of selected species involved in the investigated reactions.

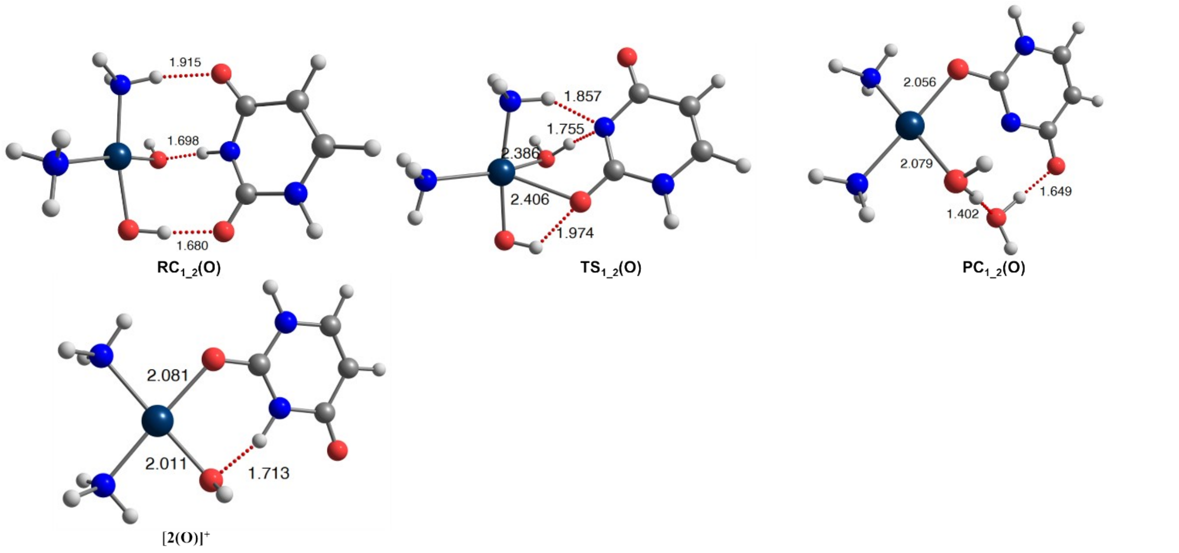

Figure S2: Optimized geometries of the transition state, reactant, and product complexes involved in the reaction between the oxygen atom of uracil in the first step of the investigated reactions.

Table S1 Unsigned Error and Mean Unsigned Error Computed for **TS4_5** Employing Different Functional with Respect to the Experimental Values

Parameter	Gas-phase (Å)	PCM-optimized (Å)	|Δ| (Å)	MUE (all bonds and)
$Pt_1 - N_{am1}$	2.098	2.095	0.003	0.021
$Pt_1 - N_{am1}$	2.085	2.086	0.001	
$Pt_1 - N_{Ur1}$	2.052	2.088	0.036	
$Pt_1 - N_{Ur2}$	2.072	2.076	0.004	
$Pt_1 - N_{am1}$	2.047	2.052	0.005	
$Pt_1 - N_{am1}$	2.047	2.056	0.009	
$Pt_1 - O_{Ur1}$	2.073	2.081	0.008	
$Pt_1 - O_{Ur2}$	2.442	2.488	0.046	
$Pt_2 - O_{wat}$	2.432	2.55	0.118	
$Pt_1 - Pt_2$	3.997	4.074	0.077	
$O_{wat} - Pt_2 - O_{Ur2}$	65°	63°	2°	2°
$Energy\ (a.u)$	-1369.755179	-1369.756201	0.001022	

Table S2 Unsigned Error and Mean Unsigned Error Computed for Pt(2.25+)$_4$ Employing Different Functional with Respect to the Experimental Values

λ (nm)	Experimental[a]	B3LYP[b]	wB97X-D[b]
1	740	790	782
2	620	593	623
3	480	485	490
**MUE**	—	**27**	**18**

Table S3: Cartesian coordinates of the species involved in the investigated reactions

1

78 -0.000001000 0.011867000 0.000017000

7 -1.488634000 1.408889000 0.000093000

7 1.488484000 1.409037000 -0.000068000

1	2.395875000	0.940947000	-0.000354000
1	1.477752000	2.014137000	-0.821163000
1	1.478094000	2.013802000	0.821279000
1	-2.395974000	0.940703000	0.000122000
1	-1.478162000	2.013833000	-0.821122000
1	-1.478102000	2.013824000	0.821314000
8	1.547613000	-1.427154000	0.000003000
8	-1.547465000	-1.427299000	-0.000153000
1	1.769239000	-1.938080000	0.787347000
1	1.769205000	-1.938113000	-0.787329000
1	-1.769043000	-1.938315000	0.787145000
1	-1.768957000	-1.938230000	-0.787531000

RC1_2

78	-1.356707000	-0.013228000	-0.034776000
7	-2.055592000	1.737116000	0.870927000
7	-2.215581000	-1.411454000	1.189381000
1	-1.865316000	-2.285111000	0.789058000
1	-1.923411000	-1.367559000	2.161230000
1	-3.230667000	-1.437082000	1.163608000
1	-1.395429000	2.086578000	1.561599000
1	-2.965878000	1.683080000	1.316739000
1	-2.119130000	2.446470000	0.143086000
8	-0.618606000	-1.705890000	-0.870105000
8	1.869371000	-2.242655000	-0.308486000
6	2.430728000	-1.086589000	-0.095444000
7	3.716655000	-1.116639000	0.339575000
6	4.362693000	0.065617000	0.611219000
6	3.730635000	1.245014000	0.454794000
6	2.361022000	1.231242000	-0.019198000
8	1.681466000	2.257473000	-0.202983000
7	1.783626000	0.021738000	-0.282569000

1	4.174297000	-2.008141000	0.454780000
1	5.386340000	-0.026202000	0.950392000
1	4.223984000	2.184012000	0.662275000
1	-0.966853000	-1.881466000	-1.746570000
1	0.909890000	-2.087153000	-0.589744000
8	-0.489776000	1.391829000	-1.322936000
1	-0.211674000	0.991489000	-2.151047000
1	0.353152000	1.779798000	-0.896214000

TS1_2

78	1.141086000	0.014106000	-0.008213000
7	1.165958000	1.906129000	-0.887809000
7	2.641890000	-0.866919000	-1.103552000
1	2.649421000	-1.816134000	-0.725539000
1	2.462728000	-0.927918000	-2.101041000
1	3.564022000	-0.464262000	-0.970089000
1	1.284787000	1.914307000	-1.895738000
1	1.873564000	2.520330000	-0.496207000
1	0.244979000	2.315095000	-0.681265000
8	1.009379000	-1.849795000	0.757546000
8	-1.391160000	-2.323829000	0.154218000
6	-1.996607000	-1.194956000	-0.008482000
7	-3.328419000	-1.290664000	-0.254587000
6	-4.085241000	-0.163723000	-0.444454000
6	-3.515764000	1.051987000	-0.366576000
6	-2.102587000	1.140694000	-0.065751000
8	-1.516204000	2.222433000	0.097160000
7	-1.387427000	-0.032659000	0.054568000
1	-3.737525000	-2.212959000	-0.275673000
1	-5.136008000	-0.327732000	-0.644681000
1	-4.086024000	1.960256000	-0.498591000
1	1.162859000	-1.856061000	1.705402000

1	-0.397403000	-2.201685000	0.421858000
8	0.298413000	1.152308000	1.908660000
1	0.870774000	1.627833000	2.514263000
1	-0.407095000	1.744515000	1.598457000

PC1_2

78	-0.820192000	-0.341335000	0.087109000
7	-1.140231000	1.221331000	1.403691000
7	-2.899889000	-0.756512000	0.031573000
1	-3.321392000	-0.916553000	0.926195000
1	-3.403425000	-0.007518000	-0.406555000
1	-3.064528000	-1.583415000	-0.511134000
1	-1.623929000	0.965756000	2.241771000
1	-1.658095000	1.943225000	0.928465000
1	-0.272706000	1.640685000	1.671511000
8	-0.541777000	-2.023727000	-1.171868000
8	1.695529000	-2.151397000	0.215880000
6	2.071614000	-0.999441000	0.147694000
7	3.405205000	-0.719801000	0.177799000
6	3.900709000	0.542854000	0.003228000
6	3.084672000	1.573740000	-0.191614000
6	1.647404000	1.340369000	-0.193041000
8	0.844479000	2.230954000	-0.351105000
7	1.211618000	0.047430000	0.023974000
1	4.010098000	-1.502332000	0.273199000
1	4.969421000	0.632638000	0.028471000
1	3.440494000	2.572329000	-0.338309000
1	-0.405559000	-1.887751000	-2.094958000
1	0.227995000	-2.462906000	-0.803793000
8	-1.967213000	2.867285000	-0.777018000
1	-2.390289000	3.639586000	-1.108112000
1	-1.044566000	2.913220000	-0.985203000

2

78	-0.960925000	0.027424000	-0.001203000
7	-0.984368000	1.914543000	0.785432000
7	-3.050725000	-0.132778000	0.009533000
1	-3.277374000	-1.100961000	-0.210522000
1	-3.476944000	0.067830000	0.910346000
1	-3.513890000	0.447171000	-0.685558000
1	-0.247134000	2.411422000	0.256247000
1	-0.723560000	1.917280000	1.767748000
1	-1.861615000	2.416334000	0.694761000
8	-1.006432000	-1.934602000	-0.750965000
8	1.149140000	-2.207879000	0.508956000
6	1.742040000	-1.142153000	0.294597000
7	3.110208000	-1.134224000	0.358487000
6	3.869317000	-0.040641000	0.044996000
6	3.278401000	1.105289000	-0.319346000
6	1.832481000	1.192396000	-0.342686000
8	1.251831000	2.239382000	-0.613971000
7	1.110265000	0.033939000	-0.016664000
1	3.534437000	-2.018781000	0.592718000
1	4.941685000	-0.172180000	0.111601000
1	3.841026000	1.991447000	-0.573467000
1	-0.827526000	-2.008247000	-1.693153000
1	-0.224400000	-2.325290000	-0.251957000

RC2_3

78	0.598298000	-0.688950000	-0.179688000
7	-0.413574000	0.957598000	-0.782303000
7	-1.191321000	-1.686893000	-0.168738000
1	-1.203808000	-2.403910000	0.549665000
1	-1.972408000	-1.008736000	0.017663000

1	-1.446248000	-2.137805000	-1.043404000
1	0.223482000	1.566697000	-1.294130000
1	-0.876365000	1.444038000	0.006828000
1	-1.183678000	0.700694000	-1.394054000
8	1.496421000	-2.476204000	0.535390000
8	3.125433000	-0.949236000	1.723141000
6	3.293188000	0.001704000	0.943365000
7	4.432026000	0.757028000	1.119519000
6	4.790699000	1.774724000	0.281139000
6	4.007168000	2.085429000	-0.757973000
6	2.761721000	1.358760000	-0.969642000
8	2.014275000	1.646596000	-1.895243000
7	2.444743000	0.333941000	-0.068763000
1	5.019088000	0.469048000	1.885083000
1	5.718832000	2.281698000	0.514207000
1	4.248824000	2.880314000	-1.448217000
1	1.954436000	-2.963760000	-0.152739000
1	2.177211000	-2.087084000	1.155388000
8	-2.294983000	2.038483000	0.995078000
6	-3.291402000	1.375074000	0.663240000
7	-4.550174000	1.901527000	0.946163000
6	-5.703291000	1.257060000	0.587438000
6	-5.657355000	0.076159000	-0.050078000
6	-4.349692000	-0.514290000	-0.345522000
8	-4.231354000	-1.605664000	-0.906086000
7	-3.230658000	0.190464000	0.035636000
1	-4.565924000	2.794560000	1.409337000
1	-6.627567000	1.759216000	0.849961000
1	-6.554936000	-0.452067000	-0.340548000

TS2_3

78	-0.031159000	-1.159841000	0.017199000

**7**	**0.632830000**	**-2.216342000**	**-1.646905000**
**7**	**-1.686335000**	**-2.329651000**	**0.416658000**
**1**	**-1.812566000**	**-2.286005000**	**1.424353000**
**1**	**-2.460388000**	**-1.817505000**	**-0.043298000**
**1**	**-1.680472000**	**-3.302298000**	**0.133937000**
**1**	**0.739875000**	**-1.458258000**	**-2.333148000**
**1**	**-0.017268000**	**-2.904766000**	**-2.006746000**
**1**	**1.530817000**	**-2.663083000**	**-1.501533000**
**8**	**-0.087658000**	**-0.842663000**	**2.448488000**
**8**	**2.485812000**	**-0.294584000**	**1.752677000**
**6**	**2.404150000**	**0.377117000**	**0.732110000**
**7**	**3.248409000**	**1.468645000**	**0.586614000**
**6**	**3.272717000**	**2.256161000**	**-0.525026000**
**6**	**2.497133000**	**1.962666000**	**-1.579408000**
**6**	**1.625614000**	**0.800032000**	**-1.518615000**
**8**	**0.993368000**	**0.413537000**	**-2.502960000**
**7**	**1.543950000**	**0.128089000**	**-0.305280000**
**1**	**3.828935000**	**1.663206000**	**1.385314000**
**1**	**3.957391000**	**3.095491000**	**-0.499460000**
**1**	**2.511529000**	**2.544371000**	**-2.489561000**
**1**	**0.870342000**	**-0.940713000**	**2.548517000**
**1**	**-0.220639000**	**0.125412000**	**2.453119000**
**8**	**-0.221984000**	**1.831555000**	**1.822540000**
**6**	**-1.116943000**	**1.806030000**	**0.968395000**
**7**	**-1.679889000**	**3.015413000**	**0.576011000**
**6**	**-2.718851000**	**3.084738000**	**-0.307861000**
**6**	**-3.239227000**	**1.961961000**	**-0.826451000**
**6**	**-2.672260000**	**0.675970000**	**-0.435875000**
**8**	**-3.170592000**	**-0.383636000**	**-0.846714000**
**7**	**-1.582518000**	**0.673212000**	**0.400165000**
**1**	**-1.310502000**	**3.837336000**	**1.023962000**
**1**	**-3.081038000**	**4.078983000**	**-0.541887000**

1	-4.066311000	1.978160000	-1.521799000

PC2_3

78	0.375850000	-0.875542000	-0.080873000
7	1.727157000	-2.414987000	-0.277817000
7	-1.238374000	-2.136821000	0.246314000
1	-1.284630000	-2.208769000	1.263519000
1	-2.082658000	-1.612673000	-0.059739000
1	-1.237819000	-3.062367000	-0.165954000
1	2.342984000	-2.076739000	-1.029247000
1	1.334606000	-3.311029000	-0.543152000
1	2.279752000	-2.548358000	0.562897000
8	-0.550951000	-0.919191000	2.935657000
8	1.343633000	1.170870000	1.983344000
6	2.023418000	1.277552000	0.970812000
7	2.936423000	2.320450000	0.876202000
6	3.810668000	2.455194000	-0.164506000
6	3.829773000	1.556412000	-1.160222000
6	2.910304000	0.428322000	-1.116373000
8	2.970134000	-0.498657000	-1.925276000
7	1.966501000	0.428368000	-0.102270000
1	2.965898000	2.937901000	1.670586000
1	4.476123000	3.309027000	-0.118494000
1	4.524862000	1.623194000	-1.984518000
1	-1.169195000	-0.447063000	3.495247000
1	0.072835000	-0.258014000	2.593544000
8	-0.787848000	0.827099000	-0.051941000
6	-2.050546000	0.950771000	-0.205730000
7	-2.475413000	2.261542000	-0.225166000
6	-3.804197000	2.564171000	-0.385413000
6	-4.708643000	1.583401000	-0.517390000
6	-4.264169000	0.182764000	-0.484820000

8	-5.042611000	-0.751527000	-0.582167000
7	-2.901187000	-0.038364000	-0.332670000
1	-1.772144000	2.973221000	-0.122569000
1	-4.049454000	3.619554000	-0.396274000
1	-5.761720000	1.792829000	-0.645008000

3

78	0.013069000	-1.100191000	0.000175000
7	-1.494896000	-2.516986000	0.300890000
7	1.529333000	-2.501390000	-0.324154000
1	1.843559000	-2.498439000	-1.288534000
1	2.282346000	-2.115180000	0.267184000
1	1.340764000	-3.459315000	-0.053534000
1	-1.823703000	-2.506278000	1.260582000
1	-1.286879000	-3.475568000	0.047205000
1	-2.245452000	-2.156376000	-0.304034000
8	-2.917705000	-0.615565000	-1.104143000
6	-2.538783000	0.337478000	-0.419753000
7	-3.312604000	1.484353000	-0.406324000
6	-3.039148000	2.561625000	0.390014000
6	-1.993440000	2.535342000	1.225353000
6	-1.120120000	1.362994000	1.261341000
8	-0.171278000	1.284126000	2.021457000
7	-1.421122000	0.333725000	0.361192000
1	-4.115795000	1.463640000	-1.011582000
1	-3.719471000	3.400468000	0.302621000
1	-1.756221000	3.362766000	1.878609000
8	0.138585000	1.373505000	-1.942211000
6	1.091123000	1.383641000	-1.184727000
7	1.919085000	2.504799000	-1.151711000
6	3.015539000	2.611227000	-0.349171000
6	3.381290000	1.587618000	0.434715000

6	2.593767000	0.365913000	0.423498000
8	2.964128000	-0.634300000	1.045659000
7	1.432430000	0.357313000	-0.333385000
1	1.640748000	3.250827000	-1.766795000
1	3.554111000	3.550167000	-0.399927000
1	4.249847000	1.628363000	1.075295000

RC3_4

78	-2.646935000	-0.681645000	-0.622038000
7	-4.281330000	-1.560059000	0.277370000
7	-3.137380000	-1.265104000	-2.578953000
1	-2.407285000	-0.915013000	-3.196108000
1	-3.172351000	-2.272307000	-2.713803000
1	-4.015602000	-0.882392000	-2.919813000
1	-3.992765000	-2.139669000	1.061762000
1	-4.871205000	-2.136753000	-0.315575000
1	-4.865134000	-0.812059000	0.649777000
8	-3.664994000	1.556545000	1.192807000
6	-2.783507000	0.959954000	1.801565000
7	-2.447765000	1.313073000	3.090903000
6	-1.428139000	0.721707000	3.780736000
6	-0.684150000	-0.242170000	3.211224000
6	-0.983936000	-0.677027000	1.861477000
8	-0.337520000	-1.536445000	1.263462000
7	-2.074464000	-0.075409000	1.243627000
1	-2.998055000	2.051992000	3.502634000
1	-1.268813000	1.080495000	4.790029000
1	0.119787000	-0.725321000	3.747261000
8	-1.021165000	0.251785000	-1.464889000
6	-0.605617000	1.399427000	-1.117551000
7	-1.462767000	2.367545000	-0.689974000
6	-1.016957000	3.621129000	-0.384203000

6	0.296623000	3.914580000	-0.475017000
6	1.177601000	2.851938000	-0.840080000
8	2.440046000	3.077129000	-0.799263000
7	0.705108000	1.647821000	-1.190862000
1	-2.439123000	2.128412000	-0.526593000
1	-1.773653000	4.328316000	-0.070085000
1	0.692232000	4.891190000	-0.237023000
78	3.262890000	-0.624132000	-0.030942000
7	2.320774000	-2.336058000	0.679137000
7	4.587114000	-0.375040000	1.505166000
1	5.008341000	0.538847000	1.332227000
1	4.173391000	-0.353057000	2.433060000
1	5.337324000	-1.061812000	1.515607000
1	1.375112000	-2.088158000	1.009629000
1	2.791727000	-2.841024000	1.423105000
1	2.192536000	-2.996689000	-0.082970000
8	4.015738000	1.117566000	-0.721292000
1	4.699248000	1.020504000	-1.387935000
1	3.060291000	2.257328000	-0.872461000
8	1.960390000	-0.709441000	-1.656276000
1	2.423035000	-0.741636000	-2.498627000
1	1.449969000	0.147631000	-1.611830000

TS3_4

78	-1.942499000	-1.051354000	-0.180627000
7	-1.551272000	-2.936529000	0.615735000
7	-3.190059000	-1.751980000	-1.704414000
1	-4.033338000	-1.176935000	-1.611562000
1	-2.815490000	-1.596012000	-2.635578000
1	-3.474549000	-2.725359000	-1.656724000
1	-1.780567000	-3.731957000	0.027136000
1	-2.054592000	-3.058578000	1.491576000
1	-0.557427000	-2.996658000	0.824366000

8	-2.893920000	0.527139000	2.196962000
6	-1.695442000	0.423102000	2.319867000
7	-1.031345000	0.998323000	3.395661000
6	0.313704000	0.956442000	3.551904000
6	1.087984000	0.329688000	2.642274000
6	0.462218000	-0.312539000	1.519767000
8	1.126682000	-0.937967000	0.646179000
7	-0.892987000	-0.257820000	1.409010000
1	-1.622913000	1.468469000	4.065327000
1	0.710329000	1.446283000	4.432712000
1	2.161888000	0.286575000	2.747367000
8	-4.556664000	0.626857000	-0.889244000
6	-3.589165000	1.349461000	-0.829621000
7	-3.721110000	2.731324000	-0.780888000
6	-2.677221000	3.584025000	-0.837379000
6	-1.408197000	3.116810000	-0.952327000
6	-1.237283000	1.709536000	-0.965069000
8	-0.092648000	1.129121000	-1.110783000
7	-2.280262000	0.880809000	-0.824341000
1	-4.671719000	3.072080000	-0.734461000
1	-2.915173000	4.640125000	-0.805607000
1	-0.561332000	3.782183000	-1.034972000
78	3.094647000	0.086926000	-0.309199000
7	3.855551000	-1.815599000	0.007968000
7	4.846490000	1.147964000	-0.106956000
1	4.593655000	2.114629000	0.090980000
1	5.429549000	0.834139000	0.663863000
1	5.421961000	1.145349000	-0.945560000
1	4.862725000	-1.945015000	0.018122000
1	3.480737000	-2.389000000	-0.746617000
1	3.484927000	-2.189489000	0.877862000
8	2.200213000	1.881301000	-0.659485000

1	2.587725000	2.324946000	-1.418424000
1	0.808215000	1.628357000	-0.928595000
8	1.597821000	-1.194063000	-1.842407000
1	1.451832000	-0.797145000	-2.703839000
1	0.789493000	-1.085306000	-1.319684000

PC3_4

78	-2.142811000	-0.837431000	-0.088353000
7	-3.289795000	-1.553524000	1.452945000
7	-2.786573000	-2.231827000	-1.506163000
1	-3.285702000	-1.774702000	-2.264440000
1	-1.936210000	-2.642386000	-1.903961000
1	-3.374390000	-2.993976000	-1.182601000
1	-2.735596000	-1.930608000	2.216813000
1	-3.984155000	-2.252884000	1.208312000
1	-3.784608000	-0.725738000	1.801606000
8	-3.448149000	1.298264000	1.801541000
6	-2.237518000	1.391699000	1.877313000
7	-1.637482000	2.399253000	2.609827000
6	-0.297239000	2.471837000	2.813592000
6	0.533767000	1.551024000	2.280516000
6	-0.032152000	0.503473000	1.490824000
8	0.615527000	-0.467988000	1.006542000
7	-1.373255000	0.509789000	1.254246000
1	-2.269404000	3.048531000	3.056671000
1	0.047564000	3.290705000	3.432712000
1	1.596249000	1.579944000	2.453331000
8	-1.191592000	0.013399000	-1.765804000
6	-0.725254000	1.214663000	-1.806330000
7	-1.432089000	2.120334000	-2.542302000
6	-1.004410000	3.408493000	-2.684594000
6	0.136363000	3.804947000	-2.079285000

6	0.838636000	2.821986000	-1.320251000
8	1.927040000	3.158453000	-0.729515000
7	0.393966000	1.562826000	-1.210040000
1	-2.287848000	1.808585000	-2.978320000
1	-1.621821000	4.056434000	-3.293153000
1	0.516735000	4.812571000	-2.166674000
78	2.548209000	-0.653083000	0.303762000
7	1.899021000	-2.611399000	0.051158000
7	4.429509000	-0.867307000	-0.480801000
1	5.072898000	-1.376124000	0.120217000
1	4.465797000	-1.292276000	-1.403032000
1	4.776469000	0.088759000	-0.568296000
1	1.334619000	-2.862044000	0.858487000
1	2.615189000	-3.324040000	-0.045637000
1	1.284246000	-2.656026000	-0.771537000
8	3.119738000	1.286993000	0.450063000
1	3.592707000	1.502778000	1.256063000
1	2.388806000	2.379893000	-0.230121000
8	0.095574000	-2.382135000	-2.247637000
1	0.403370000	-2.613121000	-3.127301000
1	-0.077330000	-1.425830000	-2.225398000

4

78	-1.952088000	-0.621273000	-0.752667000
7	-1.918499000	-2.646220000	-1.235021000
7	-3.282470000	-0.122437000	-2.272938000
1	-4.085328000	0.295977000	-1.791966000
1	-2.901764000	0.575622000	-2.905877000
1	-3.627901000	-0.887234000	-2.845872000
1	-0.980801000	-3.027054000	-1.147992000
1	-2.254683000	-2.898835000	-2.160691000
1	-2.507772000	-3.128876000	-0.557304000

8	-2.688946000	-1.797074000	1.903261000
6	-1.521767000	-1.495441000	1.988515000
7	-0.855770000	-1.526447000	3.206357000
6	0.466972000	-1.271210000	3.335547000
6	1.218605000	-0.984783000	2.250836000
6	0.582875000	-0.954375000	0.970052000
8	1.233734000	-0.831663000	-0.125368000
7	-0.759070000	-1.096629000	0.888860000
1	-1.417997000	-1.798934000	4.000658000
1	0.874914000	-1.327658000	4.337078000
1	2.278868000	-0.796064000	2.332173000
8	-4.417556000	1.260175000	-0.060836000
6	-3.388384000	1.781232000	0.307352000
7	-3.413088000	2.896363000	1.135471000
6	-2.308161000	3.580830000	1.504360000
6	-1.087201000	3.203611000	1.055866000
6	-1.028323000	2.059634000	0.215946000
8	0.047688000	1.629607000	-0.335706000
7	-2.123180000	1.333704000	-0.056350000
1	-4.334411000	3.194972000	1.426420000
1	-2.462500000	4.435955000	2.150615000
1	-0.192586000	3.755235000	1.305217000
78	3.099350000	0.034212000	-0.275272000
7	3.661845000	-1.791529000	-1.123014000
7	4.946987000	0.941700000	-0.540053000
1	5.317619000	1.306282000	0.335617000
1	5.665429000	0.323298000	-0.909347000
1	4.882380000	1.723760000	-1.189275000
1	4.075238000	-1.732268000	-2.050527000
1	2.804035000	-2.331477000	-1.219352000
1	4.291972000	-2.339551000	-0.541416000
8	2.345024000	1.706810000	0.576027000

1	2.925660000	2.464300000	0.663070000
1	0.967845000	1.890110000	0.052359000

RC4_5

78	1.298429000	-0.860238000	-0.742095000
7	0.298604000	-1.385736000	-2.490245000
7	2.143544000	-2.750092000	-0.505676000
1	3.150116000	-2.552794000	-0.469552000
1	1.894649000	-3.194231000	0.373610000
1	1.978205000	-3.429630000	-1.241445000
1	-0.643951000	-1.002391000	-2.404956000
1	0.216615000	-2.378405000	-2.687514000
1	0.740501000	-0.958540000	-3.300774000
8	2.729540000	1.864638000	-0.984383000
6	1.544034000	2.077197000	-1.025414000
7	1.051292000	3.376704000	-1.090392000
6	-0.260739000	3.683354000	-1.138121000
6	-1.188524000	2.697594000	-1.131572000
6	-0.740551000	1.347180000	-1.088283000
8	-1.574526000	0.364563000	-1.196801000
7	0.574266000	1.067945000	-0.999747000
1	1.757543000	4.099063000	-1.120220000
1	-0.509915000	4.735993000	-1.193802000
1	-2.243166000	2.917055000	-1.196411000
8	4.265601000	-1.093052000	0.051630000
6	3.724503000	-0.337313000	0.831084000
7	4.476423000	0.459744000	1.669257000
6	3.944023000	1.267347000	2.612964000
6	2.604519000	1.316086000	2.784194000
6	1.794201000	0.531500000	1.920833000
8	0.517653000	0.614094000	2.097312000
7	2.335236000	-0.227420000	0.950948000

1	5.476363000	0.393220000	1.539004000
1	4.641983000	1.837346000	3.213166000
1	2.137033000	1.918269000	3.548229000
78	-2.790488000	-0.207716000	0.387131000
7	-4.399300000	0.654880000	-0.634196000
7	-3.831161000	-0.884471000	2.018729000
1	-4.284573000	-0.157023000	2.565377000
1	-4.525286000	-1.600046000	1.819247000
1	-3.110784000	-1.308573000	2.605647000
1	-5.187917000	0.026817000	-0.767184000
1	-4.080182000	0.918217000	-1.564304000
1	-4.762586000	1.496228000	-0.193299000
8	-1.230001000	-1.033884000	1.357879000
1	-0.899746000	-1.774421000	0.842947000
1	-0.101548000	-0.095185000	1.689419000

TS4_5

78	1.417594000	0.274570000	-1.011903000
7	1.030766000	1.756003000	-2.446759000
7	2.559675000	-0.907362000	-2.295750000
1	3.433701000	-1.003959000	-1.762327000
1	2.200813000	-1.845267000	-2.447585000
1	2.785532000	-0.522022000	-3.207451000
1	0.040892000	1.991337000	-2.407040000
1	1.240609000	1.518574000	-3.411901000
1	1.553520000	2.603470000	-2.237743000
8	2.583602000	1.681463000	1.416564000
6	1.432985000	2.023242000	1.341960000
7	0.898647000	2.957432000	2.228731000
6	-0.375454000	3.391441000	2.187114000
6	-1.233221000	2.892002000	1.263192000
6	-0.746760000	1.914913000	0.349494000

8	-1.532137000	1.417581000	-0.556866000
7	0.539420000	1.533594000	0.380058000
1	1.552533000	3.320013000	2.909217000
1	-0.657195000	4.140548000	2.917072000
1	-2.252008000	3.242379000	1.193540000
8	4.017126000	-1.003818000	0.069054000
6	3.096778000	-1.293740000	0.808933000
7	3.335071000	-1.874227000	2.038142000
6	2.348562000	-2.335169000	2.846547000
6	1.056155000	-2.244174000	2.470191000
6	0.739496000	-1.622382000	1.215015000
8	-0.446829000	-1.584236000	0.793415000
7	1.762134000	-1.096186000	0.476414000
1	4.308712000	-1.980156000	2.284597000
1	2.665126000	-2.784282000	3.779693000
1	0.257271000	-2.629703000	3.085857000
78	-2.405177000	-0.394709000	-0.052647000
7	-4.070778000	0.728369000	0.342013000
7	-3.182422000	-2.227616000	0.425493000
1	-2.486187000	-2.653400000	1.039022000
1	-4.086560000	-2.280375000	0.884873000
1	-3.220789000	-2.793965000	-0.420266000
1	-4.923710000	0.359286000	-0.070750000
1	-3.908174000	1.643637000	-0.074895000
1	-4.239938000	0.864746000	1.335434000
8	-1.321699000	-1.861369000	-1.662746000
1	-1.181840000	-1.519067000	-2.548302000
1	-0.470169000	-1.944740000	-1.214597000

5

78	0.890314000	0.111833000	-1.132510000

7	0.597070000	1.594040000	-2.581667000
7	1.285267000	-1.352623000	-2.567792000
1	2.200311000	-1.716986000	-2.276176000
1	0.628541000	-2.126265000	-2.549955000
1	1.361377000	-1.046380000	-3.532776000
1	0.002035000	2.337477000	-2.225429000
1	0.178582000	1.280487000	-3.452816000
1	1.489817000	2.023241000	-2.818547000
8	2.948062000	1.528670000	0.601822000
6	1.881957000	2.036103000	0.846484000
7	1.768293000	3.133339000	1.691520000
6	0.593710000	3.731875000	1.993872000
6	-0.568945000	3.257956000	1.487134000
6	-0.509077000	2.115659000	0.641836000
8	-1.599033000	1.619644000	0.172961000
7	0.678803000	1.579950000	0.301762000
1	2.639412000	3.472747000	2.075372000
1	0.643871000	4.581100000	2.664357000
1	-1.525215000	3.693413000	1.734073000
8	3.272821000	-1.814059000	-0.713669000
6	2.628254000	-1.756027000	0.313426000
7	3.145385000	-2.242950000	1.495484000
6	2.442966000	-2.297064000	2.652179000
6	1.162273000	-1.872722000	2.689295000
6	0.590914000	-1.329459000	1.496330000
8	-0.628568000	-0.956262000	1.559625000
7	1.337751000	-1.224752000	0.374709000
1	4.093983000	-2.586688000	1.440651000
1	2.958792000	-2.706074000	3.511856000
1	0.566357000	-1.915672000	3.588434000
78	-2.080155000	-0.381137000	0.231950000
7	-3.692785000	0.288126000	-0.870730000

7	-2.731483000	-2.331922000	0.446205000
1	-2.128554000	-2.772534000	1.139609000
1	-3.680483000	-2.402632000	0.806002000
1	-2.686205000	-2.896213000	-0.397880000
1	-3.759038000	-0.085025000	-1.813605000
1	-3.582639000	1.299037000	-0.946614000
1	-4.589975000	0.128292000	-0.418572000

Solvent optimised Species

1

78	-0.000026000	0.010973000	0.000019000
7	-1.470419000	1.409487000	0.005635000
7	1.468906000	1.410946000	-0.005719000
1	2.368886000	0.956819000	-0.142635000
1	1.361834000	2.097454000	-0.747136000
1	1.518724000	1.915304000	0.875418000
1	-2.370079000	0.954298000	0.141129000
1	-1.519886000	1.914767000	-0.874994000
1	-1.364759000	2.095262000	0.747937000
8	1.569713000	-1.413513000	-0.006706000
8	-1.568062000	-1.415248000	0.006601000
1	1.654303000	-1.921567000	0.806382000
1	1.559832000	-2.028022000	-0.747564000
1	-1.557739000	-2.029462000	0.747712000
1	-1.651720000	-1.923711000	-0.806327000

RC1_2

78	1.363511000	0.004715000	0.030871000
7	2.164068000	1.534143000	-1.111576000
7	2.201984000	-1.486705000	-1.065319000

1	1.925818000	-2.358281000	-0.617289000
1	1.878208000	-1.510453000	-2.027486000
1	3.216819000	-1.455873000	-1.082831000
1	1.788628000	1.553299000	-2.055162000
1	3.175043000	1.485692000	-1.191975000
1	1.943780000	2.429260000	-0.683211000
8	0.599607000	-1.580005000	1.086411000
8	-1.728129000	-2.185696000	0.392964000
6	-2.379935000	-1.089797000	0.144263000
7	-3.594957000	-1.229444000	-0.448883000
6	-4.323585000	-0.115217000	-0.767240000
6	-3.835159000	1.116295000	-0.513685000
6	-2.526549000	1.224253000	0.097896000
8	-1.952909000	2.310920000	0.317736000
7	-1.881777000	0.074743000	0.445553000
1	-3.937323000	-2.153967000	-0.663095000
1	-5.283459000	-0.298207000	-1.230668000
1	-4.395347000	2.004063000	-0.772542000
1	0.689656000	-1.447220000	2.032064000
1	-0.760171000	-1.937955000	0.728272000
8	0.519820000	1.586111000	1.164041000
1	0.461383000	1.377676000	2.099974000
1	-0.397899000	1.831230000	0.860937000

TS1_2

78	1.127564000	0.000605000	-0.016753000
7	1.139084000	1.820465000	-0.930314000
7	2.560220000	-0.750337000	-1.278883000
1	2.751436000	-1.712517000	-1.012226000
1	2.264399000	-0.761341000	-2.250034000
1	3.437539000	-0.241717000	-1.229227000
1	1.204907000	1.769863000	-1.942212000

1	1.902286000	2.407805000	-0.606918000
1	0.256540000	2.286303000	-0.697285000
8	1.035342000	-1.907252000	0.846131000
8	-1.432160000	-2.318501000	0.264438000
6	-2.009386000	-1.227268000	0.059739000
7	-3.327382000	-1.260681000	-0.343460000
6	-4.038261000	-0.124848000	-0.588986000
6	-3.465601000	1.082725000	-0.419098000
6	-2.092704000	1.132757000	0.032420000
8	-1.513004000	2.223802000	0.264994000
7	-1.412613000	-0.033298000	0.216202000
1	-3.753465000	-2.168351000	-0.449828000
1	-5.061224000	-0.266693000	-0.912270000
1	-4.008979000	1.999685000	-0.597923000
1	1.219666000	-1.899597000	1.789592000
1	0.076266000	-2.184384000	0.700597000
8	0.402614000	1.198785000	1.942364000
1	1.068252000	1.813223000	2.263012000
1	-0.329430000	1.722584000	1.558741000

PC1_2

78	-0.856558000	-0.320047000	0.060327000
7	-1.135253000	1.223125000	1.367409000
7	-2.890452000	-0.739659000	0.100535000
1	-3.239717000	-0.945748000	1.031672000
1	-3.454530000	0.018847000	-0.271612000
1	-3.080061000	-1.559036000	-0.470053000
1	-1.738706000	0.975459000	2.145190000
1	-1.548125000	2.017882000	0.864090000
1	-0.258135000	1.545675000	1.762938000
8	-0.622507000	-2.014265000	-1.187386000
8	1.587414000	-2.234637000	0.214605000

6	2.004810000	-1.069559000	0.157197000
7	3.354120000	-0.841620000	0.256780000
6	3.912120000	0.393714000	0.114568000
6	3.133600000	1.458916000	-0.136277000
6	1.699584000	1.286988000	-0.235321000
8	0.951242000	2.237236000	-0.489060000
7	1.188155000	0.015519000	-0.013777000
1	3.932946000	-1.656612000	0.388974000
1	4.988919000	0.441566000	0.208233000
1	3.547541000	2.448121000	-0.266352000
1	-0.463698000	-1.796175000	-2.110656000
1	0.198443000	-2.408073000	-0.812319000
8	-1.642710000	3.126401000	-0.677092000
1	-1.658729000	4.083729000	-0.622251000
1	-0.718842000	2.858270000	-0.819549000

2

78	-0.966017000	0.017420000	-0.001990000
7	-0.993424000	1.849836000	0.887229000
7	-3.040963000	-0.073110000	0.009166000
1	-3.340899000	-0.995191000	-0.295544000
1	-3.442646000	0.076320000	0.930245000
1	-3.467625000	0.599981000	-0.620934000
1	-0.325404000	2.432831000	0.378570000
1	-0.691298000	1.788984000	1.855058000
1	-1.899465000	2.306376000	0.881299000
8	-1.001551000	-1.934563000	-0.806550000
8	1.186406000	-2.194953000	0.546788000
6	1.767765000	-1.124355000	0.310332000
7	3.138393000	-1.098753000	0.373122000
6	3.878473000	0.002525000	0.062617000
6	3.267257000	1.135314000	-0.317221000

6	1.820965000	1.183311000	-0.378444000
8	1.222846000	2.208180000	-0.720181000
7	1.120182000	0.032535000	-0.021213000
1	3.585846000	-1.969068000	0.616012000
1	4.951518000	-0.109828000	0.141434000
1	3.823225000	2.025809000	-0.571960000
1	-0.866147000	-1.961584000	-1.758504000
1	-0.215431000	-2.337006000	-0.362765000

RC2_3

78	0.606599000	-0.777481000	-0.239310000
7	-0.160812000	0.613518000	-1.501324000
7	-1.166032000	-1.809427000	-0.425265000
1	-1.256633000	-2.502782000	0.310901000
1	-1.967470000	-1.153474000	-0.347726000
1	-1.246287000	-2.304786000	-1.308207000
1	0.599547000	1.119872000	-1.949585000
1	-0.790484000	1.249084000	-0.983073000
1	-0.709310000	0.183837000	-2.239638000
8	1.236916000	-2.255329000	1.147229000
8	2.436145000	-0.271179000	2.272815000
6	2.903099000	0.359358000	1.309236000
7	4.004147000	1.149787000	1.530603000
6	4.637295000	1.838873000	0.540262000
6	4.181593000	1.765833000	-0.719699000
6	3.008016000	0.966733000	-1.013920000
8	2.557331000	0.883675000	-2.159937000
7	2.390758000	0.304473000	0.045028000
1	4.356438000	1.161838000	2.475057000
1	5.499497000	2.419359000	0.840729000
1	4.659725000	2.296309000	-1.530373000
1	1.837137000	-2.901255000	0.764348000

1	1.737950000	-1.701068000	1.794996000
8	-2.038764000	2.122320000	-0.032951000
6	-3.112919000	1.505002000	0.119836000
7	-4.209601000	2.232138000	0.565484000
6	-5.431822000	1.657991000	0.754452000
6	-5.605893000	0.346275000	0.512033000
6	-4.465495000	-0.438892000	0.051122000
8	-4.570282000	-1.657167000	-0.177678000
7	-3.263694000	0.193900000	-0.124737000
1	-4.069287000	3.214684000	0.737547000
1	-6.217590000	2.317634000	1.101274000
1	-6.565304000	-0.132011000	0.655649000

TS2_3

78	0.095908000	-1.145953000	0.001947000
7	0.964925000	-2.104574000	-1.603219000
7	-1.367079000	-2.572987000	0.282534000
1	-1.526157000	-2.692286000	1.278111000
1	-2.210074000	-2.159928000	-0.135811000
1	-1.207349000	-3.491757000	-0.115428000
1	0.947510000	-1.434904000	-2.371021000
1	0.490954000	-2.948045000	-1.907679000
1	1.932067000	-2.349430000	-1.419136000
8	0.010328000	-0.814170000	2.447405000
8	2.558382000	-0.127968000	1.723597000
6	2.438442000	0.562772000	0.707500000
7	3.289878000	1.634503000	0.532404000
6	3.262401000	2.440902000	-0.564948000
6	2.387613000	2.201430000	-1.555564000
6	1.484210000	1.071968000	-1.450221000
8	0.725743000	0.753424000	-2.371786000
7	1.510361000	0.338579000	-0.269539000

1	3.960501000	1.790357000	1.268150000
1	3.980425000	3.250629000	-0.576622000
1	2.353951000	2.812978000	-2.445800000
1	0.978797000	-0.773322000	2.485529000
1	-0.271676000	0.124633000	2.436316000
8	-0.621925000	1.772357000	1.970450000
6	-1.458062000	1.671067000	1.052364000
7	-2.108187000	2.821281000	0.639127000
6	-3.089286000	2.800154000	-0.306545000
6	-3.457336000	1.634578000	-0.867660000
6	-2.784420000	0.412284000	-0.455613000
8	-3.132254000	-0.692261000	-0.918157000
7	-1.761352000	0.501936000	0.453237000
1	-1.855966000	3.679766000	1.102752000
1	-3.536039000	3.755705000	-0.550162000
1	-4.241184000	1.586746000	-1.610790000

PC2_3

78	0.320438000	-0.969667000	0.225846000
7	1.710040000	-2.394975000	-0.251132000
7	-1.248763000	-2.325880000	0.380599000
1	-1.478589000	-2.530610000	1.346758000
1	-2.054373000	-1.859933000	-0.052910000
1	-1.101453000	-3.211740000	-0.089007000
1	2.033324000	-2.189171000	-1.195792000
1	1.385059000	-3.356993000	-0.233937000
1	2.513846000	-2.339518000	0.366987000
8	-0.503416000	0.191379000	3.500463000
8	1.959733000	0.797115000	2.301840000
6	2.318563000	1.046486000	1.149884000
7	3.274625000	2.024804000	0.951794000
6	3.785128000	2.334664000	-0.270924000

6	3.369877000	1.676917000	-1.365273000
6	2.371149000	0.633218000	-1.232513000
8	1.992663000	-0.045702000	-2.191202000
7	1.846596000	0.409862000	0.036312000
1	3.606702000	2.491235000	1.780938000
1	4.532722000	3.117144000	-0.288229000
1	3.771031000	1.891791000	-2.345053000
1	-1.042338000	0.302763000	2.708433000
1	0.400517000	0.337552000	3.176040000
8	-0.997988000	0.592015000	0.687227000
6	-2.119924000	0.813246000	0.102863000
7	-2.631218000	2.065679000	0.320034000
6	-3.820874000	2.443240000	-0.237816000
6	-4.506422000	1.578251000	-1.005061000
6	-3.967814000	0.239053000	-1.221703000
8	-4.564602000	-0.600632000	-1.903453000
7	-2.757323000	-0.070801000	-0.635251000
1	-2.093119000	2.704785000	0.883853000
1	-4.145228000	3.453155000	-0.022616000
1	-5.449207000	1.853809000	-1.457468000

3

78	0.014641000	-1.075439000	-0.005688000
7	-1.472754000	-2.514178000	0.212816000
7	1.522183000	-2.483456000	-0.280126000
1	1.901567000	-2.455663000	-1.221079000
1	2.263464000	-2.208791000	0.365186000
1	1.273577000	-3.447337000	-0.086109000
1	-1.868691000	-2.507740000	1.147461000
1	-1.201549000	-3.470478000	0.010828000
1	-2.211833000	-2.252253000	-0.438887000
8	-2.694216000	-0.441116000	-1.448309000

6	-2.476572000	0.416878000	-0.589734000
7	-3.327637000	1.501384000	-0.494617000
6	-3.200836000	2.467959000	0.458544000
6	-2.221877000	2.388009000	1.373610000
6	-1.297300000	1.265755000	1.341688000
8	-0.421886000	1.121325000	2.195596000
7	-1.441396000	0.358290000	0.300015000
1	-4.084589000	1.530299000	-1.158926000
1	-3.930390000	3.266843000	0.423095000
1	-2.106191000	3.133827000	2.147203000
8	0.340133000	1.288614000	-2.061541000
6	1.239827000	1.339210000	-1.229733000
7	2.118313000	2.412157000	-1.252709000
6	3.173320000	2.537586000	-0.402514000
6	3.414435000	1.588159000	0.517045000
6	2.539951000	0.433645000	0.594474000
8	2.762019000	-0.493649000	1.385026000
7	1.448007000	0.395855000	-0.256123000
1	1.949412000	3.102535000	-1.966799000
1	3.781265000	3.424598000	-0.527560000
1	4.250885000	1.653398000	1.198007000

3(M062X)

78	0.012368000	-1.128199000	0.002190000
7	-1.500305000	-2.552656000	0.330455000
7	1.532379000	-2.536035000	-0.355832000
1	1.856577000	-2.488358000	-1.317107000
1	2.283869000	-2.184721000	0.255454000
1	1.338285000	-3.506692000	-0.135617000
1	-1.843271000	-2.498071000	1.285020000
1	-1.287796000	-3.523106000	0.126570000
1	-2.245557000	-2.224180000	-0.296636000
8	-2.879763000	-0.646804000	-1.129705000
6	-2.517504000	0.308749000	-0.442267000
7	-3.289510000	1.459000000	-0.450486000
6	-3.009619000	2.553522000	0.322338000
6	-1.963154000	2.543181000	1.158048000
6	-1.088795000	1.368949000	1.211225000
8	-0.115656000	1.317832000	1.941121000
7	-1.418556000	0.305623000	0.364199000
1	-4.078160000	1.437474000	-1.077566000
1	-3.685154000	3.393246000	0.214636000
1	-1.718101000	3.384898000	1.788622000
8	0.074968000	1.405859000	-1.847632000
6	1.053752000	1.390330000	-1.125124000
7	1.882163000	2.512791000	-1.075867000
6	2.988146000	2.600604000	-0.281235000
6	3.367019000	1.558453000	0.472122000
6	2.575139000	0.335899000	0.444341000
8	2.930539000	-0.666562000	1.067962000
7	1.426487000	0.331727000	-0.330348000
1	1.577073000	3.284909000	-1.647520000
1	3.522642000	3.542274000	-0.312351000
1	4.238942000	1.584693000	1.107823000

3(PBE0)

78	0.012443000	-1.075312000	-0.000104000
7	-1.487207000	-2.485970000	0.242621000
7	1.518495000	-2.473536000	-0.267511000
1	1.850097000	-2.502351000	-1.225712000
1	2.260971000	-2.069404000	0.332949000
1	1.317706000	-3.421392000	0.029082000
1	-1.832566000	-2.508845000	1.196372000
1	-1.268384000	-3.434490000	-0.038932000
1	-2.226676000	-2.106564000	-0.369737000
8	-2.894580000	-0.620876000	-1.167778000
6	-2.546956000	0.326751000	-0.455906000
7	-3.362669000	1.443063000	-0.412345000
6	-3.138584000	2.499443000	0.420607000
6	-2.090167000	2.487440000	1.257385000
6	-1.170021000	1.355821000	1.254755000
8	-0.215902000	1.287901000	2.010974000
7	-1.432350000	0.343854000	0.323390000
1	-4.168481000	1.403085000	-1.013990000
1	-3.853542000	3.312819000	0.360959000
1	-1.889004000	3.301806000	1.939727000
8	0.188113000	1.379185000	-1.932461000
6	1.140590000	1.375264000	-1.174378000
7	2.010269000	2.465362000	-1.172416000
6	3.110334000	2.552767000	-0.379488000
6	3.436851000	1.536738000	0.436385000
6	2.605713000	0.349495000	0.459433000
8	2.941123000	-0.649203000	1.108953000
7	1.445516000	0.363346000	-0.295508000
1	1.758392000	3.201693000	-1.810981000
1	3.688810000	3.466703000	-0.460803000
1	4.311115000	1.560036000	1.071288000

3(GD3BJ)

78	0.015096000	-1.103473000	0.001168000
7	-1.491762000	-2.527649000	0.306779000
7	1.534817000	-2.507053000	-0.328021000
1	1.824920000	-2.530499000	-1.301181000
1	2.297902000	-2.096818000	0.240752000
1	1.351689000	-3.457943000	-0.024849000
1	-1.798764000	-2.542550000	1.275133000
1	-1.284217000	-3.479349000	0.022329000
1	-2.252955000	-2.148248000	-0.278008000
8	-2.943283000	-0.646317000	-1.071522000
6	-2.554184000	0.320332000	-0.401485000
7	-3.334131000	1.469708000	-0.392144000
6	-3.051827000	2.560704000	0.385801000
6	-1.987597000	2.548602000	1.205615000
6	-1.105948000	1.383993000	1.242696000
8	-0.132288000	1.321097000	1.981122000
7	-1.424341000	0.332931000	0.364329000
1	-4.149013000	1.436730000	-0.984159000
1	-3.735311000	3.396595000	0.297160000
1	-1.743061000	3.386657000	1.842122000
8	0.095652000	1.406322000	-1.901538000
6	1.069780000	1.402255000	-1.164828000
7	1.902110000	2.528129000	-1.128661000
6	3.017907000	2.620863000	-0.348165000
6	3.399843000	1.579002000	0.412325000
6	2.612589000	0.358976000	0.400533000
8	2.994041000	-0.655245000	1.006943000
7	1.434052000	0.360719000	-0.337787000
1	1.608854000	3.286327000	-1.724584000
1	3.557530000	3.559228000	-0.394842000

1	4.283343000	1.608037000	1.032518000

TS4_5 (optimised in solvent at wB97X-D/SDD/6-311G+(d) level of theory)

78	1.485860000	0.370539000	-0.930635000
7	1.123013000	1.893965000	-2.322331000
7	2.632182000	-0.745010000	-2.271149000
1	3.458822000	-1.018085000	-1.726350000
1	2.168461000	-1.591966000	-2.603043000
1	2.952958000	-0.232270000	-3.093260000
1	1.648620000	2.741771000	-2.100866000
1	0.128884000	2.130834000	-2.293976000
1	1.344172000	1.650509000	-3.288824000
8	2.578023000	1.861575000	1.554345000
6	1.380522000	2.105980000	1.472049000
7	0.770387000	2.953678000	2.397306000
6	-0.533134000	3.319177000	2.345635000
6	-1.334912000	2.821574000	1.364843000
6	-0.771593000	1.905420000	0.423446000
8	-1.502206000	1.410229000	-0.536227000
7	0.543423000	1.580720000	0.486242000
1	1.383069000	3.323163000	3.115730000
1	-0.873682000	4.007615000	3.109651000
1	-2.374359000	3.105601000	1.288138000
8	4.080766000	-0.946463000	0.248143000
6	3.094000000	-1.394665000	0.841325000
7	3.264729000	-2.220116000	1.945346000
6	2.236028000	-2.858035000	2.572983000
6	0.967466000	-2.700400000	2.123965000
6	0.727740000	-1.846336000	0.985714000
8	-0.429836000	-1.776757000	0.473230000
7	1.786930000	-1.141699000	0.459836000
1	4.224005000	-2.385099000	2.225369000
1	2.504524000	-3.487830000	3.412692000
1	0.131717000	-3.216510000	2.576454000
78	-2.425819000	-0.401835000	-0.092121000
7	-4.122899000	0.641467000	0.401742000
7	-3.277909000	-2.228104000	0.319445000
1	-2.514236000	-2.834310000	0.634168000
1	-3.989233000	-2.220049000	1.052442000
1	-3.696309000	-2.649618000	-0.512739000
1	-4.960413000	0.237410000	-0.022530000
1	-4.036936000	1.597575000	0.049536000
1	-4.274848000	0.689446000	1.411433000
8	-1.240796000	-1.559431000	-2.031982000
1	-0.850528000	-0.784044000	-2.470826000
1	-0.581249000	-1.796919000	-1.343097000
